# High-performance multi-functional reverse osmosis membranes obtained by carbon nanotube·polyamide nanocomposite

**DOI:** 10.1038/srep13562

**Published:** 2015-09-03

**Authors:** Shigeki Inukai, Rodolfo Cruz-Silva, Josue Ortiz-Medina, Aaron Morelos-Gomez, Kenji Takeuchi, Takuya Hayashi, Akihiko Tanioka, Takumi Araki, Syogo Tejima, Toru Noguchi, Mauricio Terrones, Morinobu Endo

**Affiliations:** 1 Global Aqua Innovation Center, Shinshu University; 4-17-1 Wakasato, Nagano 380-8553, Japan; 2 Institute of Carbon Science and Technology, Shinshu University; 4-17-1 Wakasato, Nagano 380-8553, Japan; 3 Research Organization for Information Science & Technology, 2-32-3, Kitashinagawa, Shinagawa-ku, Tokyo, 140-0001; 4 Department of Physics, Department of Materials Science and Engineering, and Department of Chemistry. The Pennsylvania State University; University Park, Pennsylvania 16802, USA

## Abstract

Clean water obtained by desalinating sea water or by purifying wastewater, constitutes a major technological objective in the so-called water century. In this work, a high-performance reverse osmosis (RO) composite thin membrane using multi-walled carbon nanotubes (MWCNT) and aromatic polyamide (PA), was successfully prepared by interfacial polymerization. The effect of MWCNT on the chlorine resistance, antifouling and desalination performances of the nanocomposite membranes were studied. We found that a suitable amount of MWCNT in PA, 15.5 wt.%, not only improves the membrane performance in terms of flow and antifouling, but also inhibits the chlorine degradation on these membranes. Therefore, the present results clearly establish a solid foundation towards more efficient large-scale water desalination and other water treatment processes.

The availability of clean water has become a global problem because of the continuously increasing costs of energy and increasing scarcity of water resources^1^. This problem has been exacerbated in recent years in the so-called century of water. By far, the RO membrane process persists as the most reliable and cost-effective water desalination technique and numerous large-scale RO plants have been constructed around the world^2^,^3^. A wide range of polymers have shown potential for fabricating desalination membranes to be used in RO^4^. However, PA-based membranes tend to exhibit the best performance in terms of selectivity, flow, chemical stability, and ease of large-scale fabrication. PA membrane technology was developed in the mid-70 s and has become the commercial benchmark in RO membranes^5^. In order to improve the membrane performances, the recent trend in polymer-based membrane research has been to investigate various types of nanocomposite films as an active layer of RO membrane, so-called nanocomposite membranes, in which these films are fabricated using a nanosized filler such as MWCNT, graphene, graphene oxide, silica, or zeolite^6^. In this regard, MWCNT·PA-based membranes have been prepared by several groups and in general, these membranes have exhibited some level of improved performance^7^,^8^,^9^,^10^,^11^,^12^. The advantages claimed for these membranes range from increased salt rejection, large fluxes, greater durability, and even antimicrobial properties.

MWCNT synthesized by catalytic chemical vapour deposition^13^,^14^ have been widely studied due to their fascinating chemical and physical properties, and among all nanocarbon materials, they can be mass-produced for commercially available applications, such as the electrode additives in high performance lithium ion batteries^15^. Interestingly, while the structure of the fully aromatic PA-based RO membrane derived from *m*-phenylendiamine (MPD)-trimesoyl chloride (TMC) is constrained due to its stoichiometry; the addition of MWCNT can significantly vary their performance due to their unique features such as dispersability diameter, length, straightness, and chemical functionalities, among many others. Therefore, although these past reports acknowledge the key role of MWCNT in aromatic PA nanocomposite membranes, still little attention has been devoted to the mechanisms related to the improvement of flow rate, selectivity and chlorine tolerance^2^. Carbon nanotubes inducing chlorine tolerance are particularly interesting because chlorine sensitivity has been recognized as a major drawback of PA-based RO membranes^16^,^17^. During long-term operation, chlorine is often added as a pre-treatment to reduce algae biofouling^18^, and is particularly needed for drinking water purification. Moreover, high-concentration short-term exposure to chlorine is also common during RO membrane backwashing. For these reasons, several studies have been carried out and the degradation mechanism of aromatic PA membranes during chlorine exposure is relatively well-known^19^,^20^. Recently, our group demonstrated that the addition of MWCNT to rubber can considerably reduce the chlorine-induced degradation of the polymer matrix^21^. Although the degradation mechanism of rubber by chlorine is different from that of PA, particularly due to the lack of hydrolysis, covalent chlorination is a common problem for both polyamide and rubber. For rubber, we found that MWCNT effectively restricted the adsorption of chlorine within the polymer matrix, thus resulting in a limited exposure of the polymer to this reactive reagent and thereby decreasing the oxidative degradation. For these reasons, we believe MWCNT are not only promising composite fillers with chlorine protective properties, but might also help to provide mechanical robustness to PA-based RO membranes.

## Results and Discussion

**Figure 1:**
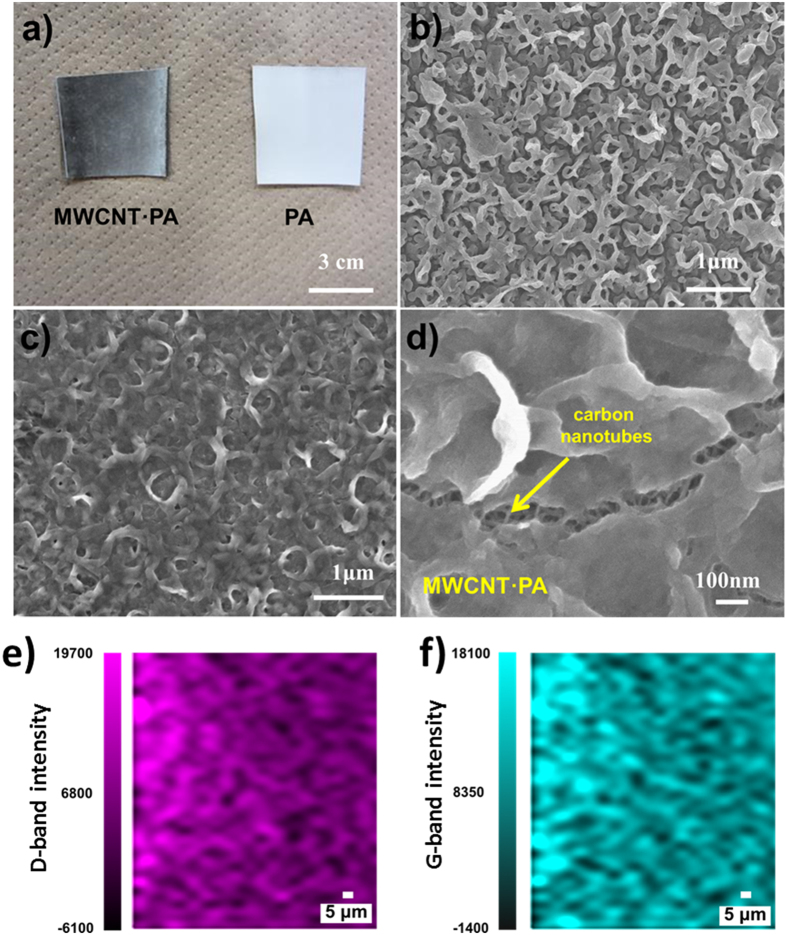
(**a**) Photographs of a plain PA and a MWCNT·PA nanocomposite RO membranes. SEM images of the surface of the (**b**) plain PA membrane and the (**c**) MWCNT·PA nanocomposite membrane. (**d**) Detail showing the reinforcing nanotubes through a fracture. Raman mapping of the nanocomposite MWCNT·PA membrane showing the intensity of the (**e**) D-band and (**f**) G-band characteristic of carbon nanotubes.

We prepared aromatic PA membranes using a support consisting of a porous polysulfone layer deposited on a polypropylene nonwoven. These support membranes were soaked sequentially in MPD and TMC solutions, to synthesize the aromatic PA membrane by interfacial polymerization. In order to incorporate MWCNT into the PA membrane, an anionically stabilized dispersion of MWCNT (Supplementary Fig. S1) was mixed with the MPD solution and the synthesis was conducted similarly. Figure 1a shows an image of the resulting membranes, with and without MWCNT. The black color developed in the membrane prepared using surfactant dispersed MWCNT is characteristic of the high carbon nanotube content of the present membrane (Fig. 1a). Thermogravimetry of the active layer (Supplementary Fig. S2) of the black color membrane indicates that it contains *ca.* 15.5 wt. % of MWCNT, which is at least 150 times higher than previously reported MWCNT-filled RO PA membranes^7^,^8^,^12^. The SEM image showing the surface morphology of the membrane is typical for the interfacial PA polymerization^22^, consisting of the multi-layered ridge-and-valley (Fig. 1b); the morphology of this membrane clearly changed after the addition of MWCNT (Fig. 1c). The thickness of the membranes was measured using SEM (Supplementary Fig. S3). The addition of MWCNT did not modify the thickness of the active layer and both samples were approximately 100 nm thick. However, water contact angle measurements showed a slight increase in wettability upon addition of MWCNT to the PA membrane (Supplementary Fig. S4). Notably, no MWCNT were visible on the surface, thus indicating that they were perfectly embedded within the PA matrix, a key factor needed for avoiding MWCNT leakage during operation. Flow permeation rates, as indicated below, and SEM images confirmed that the membranes can be produced pinhole-free in a reproducible way. After the membrane was dried for SEM studies, cracks were generated by manual deformation of the membrane (Fig. 1d), and MWCNT embedded, parallel along the membrane surface, were observed bridging the fracture within the polymer matrix. The apparent diameter of these nanotubes are *ca.* 20 nm, which is about two times larger than the pristine nanotubes (Fig. S1a). These facts suggest that the nanotubes must be coated with polymer to achieve a good matrix-nanotube adhesion. In order to support our proposed structure consisting of a polymeric network with aromatic moieties in parallel arrangement to the MWCNT walls, we performed theoretical simulations of the monomer molecules orientation in the vicinity of a carbon nanotube surface, see Supplementary Fig. S5. Here, four different cases, consisting of two geometrical configurations, are demonstrated: horizontal and vertical alignments with respect to the MWCNT surface (modelled as a graphene surface), for both monomers (MPD and TMC). The results indicate a clear energetic preference for the horizontal arrangements of these molecules interacting with sp^2^ hybridized carbon networks; these preferences are related to π-π stacking and are known to be common for aromatic compounds on sp^2^ hybridized carbon surfaces. Similarly, Fig. S5b shows a simulation of 50 MPD molecules absorbed on a graphene surface, and it can be seen that the molecules adopt a similar geometrical orientation after relaxation (Fig. S5c). In order to rule out curvature effects, we carried the simulations using a (10,10) single-walled carbon nanotube (Fig. S5d), which evidently has a higher curvature than the 10 nm diameter MWCNT experimentally used in the membrane fabrication. It can be seen on Fig. S5e that after relaxation, the aromatic ring of the MPD molecules lies parallel to the carbon nanotube surface. We confirmed the strong affinity of MPD with MWCNT by filtering the solution and carrying out UV-Vis spectroscopy. We found that 16.7% of the MPD monomer remained attached to the MWCNT. These MPD functionalized MWCNT were polymerized in TMC solution. Supplementary Fig. S6a shows a homogeneous PA coating on the MWCNT. Supplementary Fig. S6b depicts a higher resolution image showing a coating of about 5 nm thick on the MWCNT surface. We used fast Fourier transformation (FFT) of the HRTEM images to analyze the orientation of the PA network, and it is clear that PA regions that do not contain MWCNT, show an anisotropic molecular arranged structure (Supplementary Fig. S6c), whereas the PA coating the nanotubes show a preferential orientation of PA molecules along the MWCNT surface (Supplementary Fig. S6d). These experiments strongly support a templating effect caused by MWCNT. To assess the distribution of the MWCNT within the membrane, a Raman mapping of the characteristic D- and G- bands of MWCNT was conducted (see Fig. 1e,f). Through all the studied areas only the D- and G- peaks could be observed, indicating a homogenous mixture and a high content of MWCNT, which is not common in these type of nanocomposites, because the MWCNT are prone to aggregation even when loading at low concentrations. Commercial RO membranes exhibited a lower contact angle; however in this case, the presence of wetting additives or a surface treatment is likely responsible for this phenomena. The method used to synthesize the MWCNT·PA nanocomposite relies on the transport of the MWCNT to the organic/aqueous interface during polymerization^23^. Indeed, the presence of a limited amount of anionic surfactant has been recently reported to improve PA membrane formation, resulting in better performance^24^. This is most likely due to a reduction of the oil/water interfacial tension, a process that in our case is also promoted by the small amount of surfactant that provides amphiphilicity to the nanotubes It is important to emphasize that we did not used covalent functionalization of MWCNT, in contrast to some previous reports^8^,^11^.

**Figure 2:**
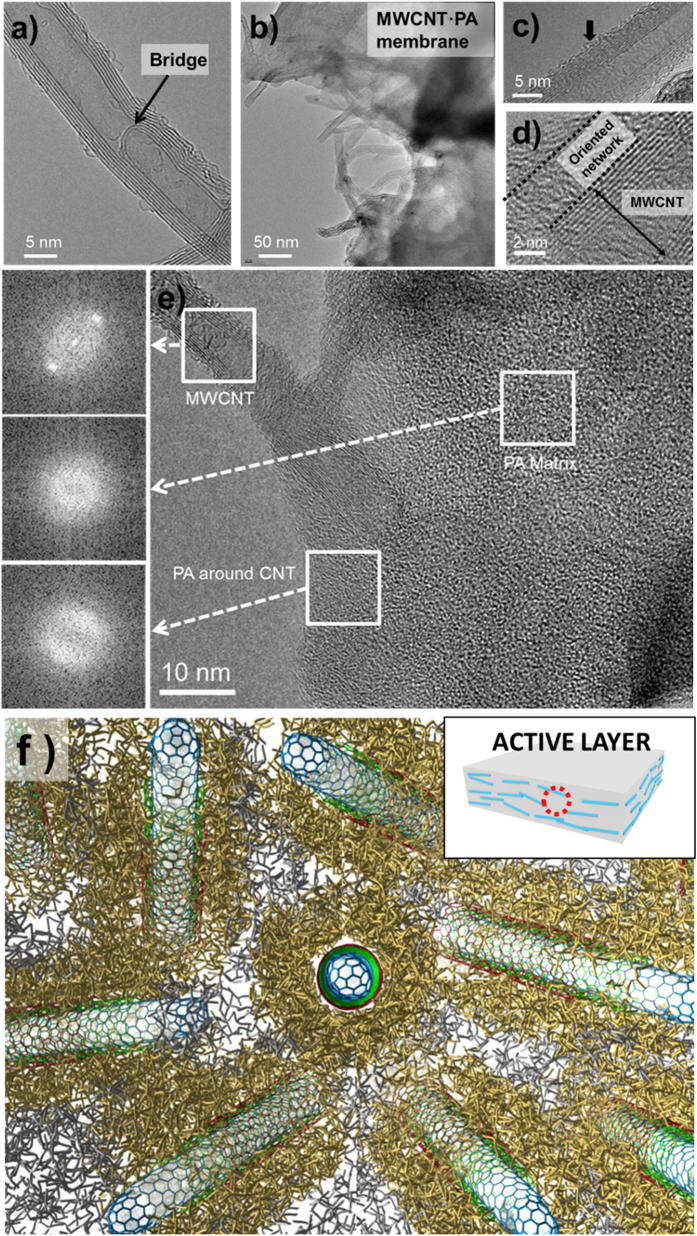
TEM images of the MWCNT·PA nanocomposite RO membranes. (**a**) shows a pristine MWCNT. (**b**) shows the border of a cleaved MWCNT·PA nanocomposite RO membrane. MWCNTs can be seen protruding from the surface. (**c**) Shows a magnification of one of these nanotubes that has been pulled out from the PA matrix. (**d**) Shows the carbon nanotube embedded within the PA matrix. In (**e**) several FFT patterns of the nanocomposite RO membranes are shown, top; corresponding to MWCNT, middle; PA zone, bottom; PA zone around MWCNT. (**f**) Model of the MWCNT·PA nanocomposite microstructure, showing the proposed ordered PA regions in yellow around MWCNT fillers.

The microstructure of the MWCNT·PA nanocomposite membrane was further studied using high-resolution transmission electron microscopy (HRTEM). Figure 2a exhibits the typical morphology of the pristine MWCNT, showing the characteristic and overall clean surface walls with a small amount of carbonaceous material deposited on the surface. Figure 2b shows the edge of the nanocomposite membrane with several MWCNT protruding from the matrix. A detail of these nanotubes (Fig. 2c) shows traces of PA attached to the surface, suggesting good interaction between the monomers and the MWCNT walls, forming an ordered region several nanometers thick, shown in Fig. 2d, in agreement with the observation of thicker diameter by SEM (Fig. 1d). Indeed, we conducted FFT of TEM images on several regions within the nanocomposite membrane (Fig. 2e), which shows a pattern that suggests order of the polymer network along the nanotubes surfaces and might represent a unique aromatic PA structure when compared to the bulk PA, as discussed above. The structured model is graphically represented in Fig. 2f. Such a characteristic ordered region has a thickness roughly similar to the nanotube diameter. The FFT pattern on the nanotube showed the typical sharp peaks caused by the concentric graphitic layers, while the FFT pattern of the matrix exhibited only a diffuse halo characteristic of disordered amorphous polymer networks.

**Figure 3:**
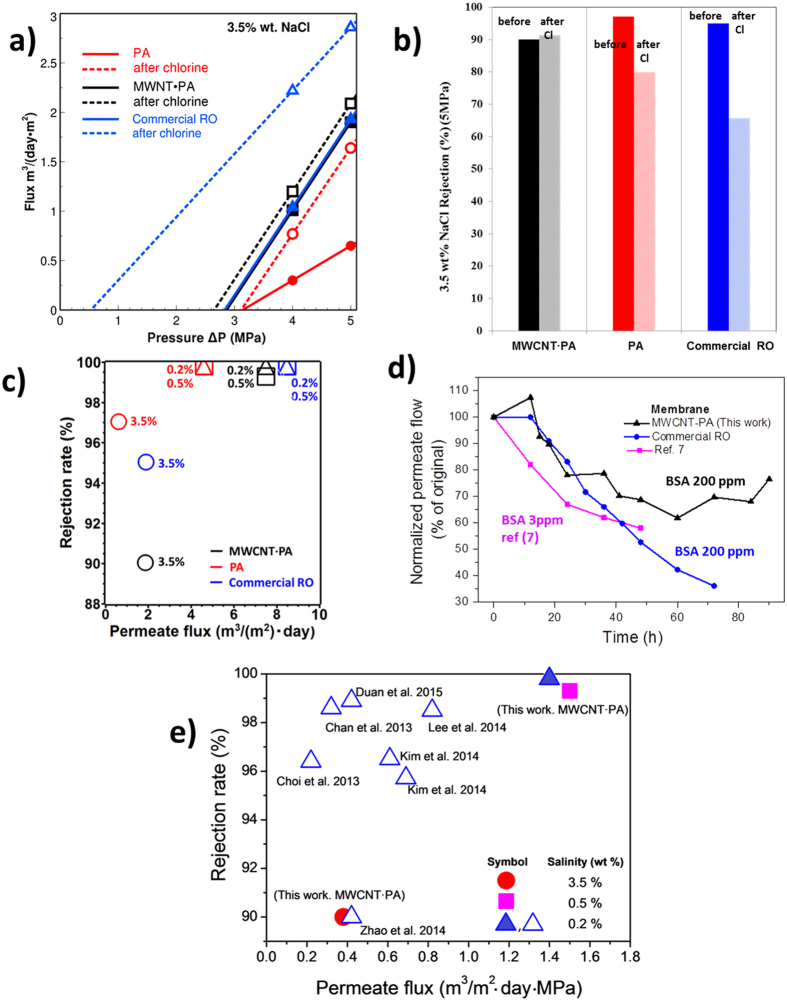
(**a**) Flow performance as a function of transmembrane pressure (ΔP, MPa) of pure PA and MWCNT·PA nanocomposite reverse osmosis membranes before and after exposure to chlorine water (200 ppm). (**b**) Salinity rejection rates before and after chlorine water exposure of the pure PA, the MWCNT·PA nanocomposite membrane, and the commercial PA RO membrane tested with 3.5 wt. % salt water. (**c**) Comparison of permeate flux/rejection performance of the PA, MWCNT·PA, and commercial RO membrane at different sodium chloride concentrations. (**d**) Fouling of the MWCNT·PA nanocomposite membrane and a commercial PA RO membrane in presence of 200 ppm BSA concentration in DI water. Data from Ref. 7 obtained with a considerable lower (3 ppm) BSA concentration is shown for comparison. (**e**) Comparison of the permeate flux/rejection performance of our membrane (solid fill symbols) with previous works.

Water flux and salt rejection of our nanocomposite membranes were evaluated by cross-flow filtration and conductivity measurements of saline water at room temperature (details in experimental part). The permeate flux plotted as a function of pressure (ΔP) for three types of membranes is shown in Fig. 3a, and the linear slope can be correlated with the corresponding membrane permeability coefficient. Indeed, the addition of MWCNT to PA dramatically increased the permeation coefficient when compared with the pure PA membrane. Consequently, the permeate flow at 5 MPa (*ca.* 1.7 m^3^/ m^2^·day) was more than double when compared to the pure PA membrane (*ca.* 0.65 m^3^/ m^2^·day), and even better performance is expected at higher pressures. We attribute this effect to the change in the molecular network structure at the MWCNT·PA ordered interface region that results in a flux-enhanced membrane, a phenomena observed in PA-based RO membranes synthesized in presence of MWCNT additives^25^. We believe there is no flow in the present MWCNT through the hollow core of the nanotubes because our CVD synthesized MWCNT commonly contains a “bridged” structure or inner compartments^26^ that interrupt the water flow through the hollow core. Since the MWCNT behave as an impermeable obstacle, the observed high permeability of the present nanocomposites must result from the newly developed PA nanostructure originated by the addition of MWCNT. When desalination of 3.5 wt.% saline water was performed, we noticed that the higher permeate flux comes at a moderate expense of the rejection rate. The pure PA membrane exhibited a 97% salt rejection rate, and the MWCNT·PA nanocomposite reached a salt rejection rate of 90 wt.% (see Fig. 3b). However, this value is sufficiently high for pre-treatment of energy-saving multi-stage desalination processes^27^, and it is similar to the rejection rates of many commercial RO membranes. In addition, when 0.5 wt.% or 0.2 wt.% saline water concentration was used (Fig. 3c), desalination rates as high as 99.8% and 99.7% respectively, were reached, thus contrasting with previous reports of nanocomposite PA-based membranes with low MWCNT content. In those reports the rejection rate decreased, even when using only 0.2 wt.% salt water^7^. Further improvements of the present membranes are possible by tuning the structure of MWCNT·PA nanocomposite, particularly if the diameter and length of MWCNT are tailored.

Chlorine exposure tests were performed by soaking the as-prepared pure PA, MWCNT·PA nanocomposite membranes and commercial PA RO membranes for comparison, in 200 ppm sodium hypochlorite solution for 24 h. Figure 3b shows that both the laboratory-synthesized PA and the commercial PA-based membranes exhibited substantially lower salt rejection, thus suggesting a change in the membrane microstructure (possibly reduced hydrogen bonding and consequently greater free-volume)^28^,^29^. In contrast, the MWCNT·PA-based nanocomposite membrane was not affected by the chlorine exposure; both the salt rejection and permeate flow values were similar before and after the chlorine water exposure. Other researchers have already added MWCNT to aromatic PA membranes in previous studies^7^,^30^,^31^ to increase the membrane chlorine resistance and in general, they attributed the protection against degradation to the electronic rich nature of the highly sp^2^-hybridized MWCNT or to sacrificial organic functionalities present on the nanotubes surface. We have previously shown that the addition of MWCNT to a polymer matrix reduced the adsorption of chlorine within the matrix, therefore protecting the polymer from the chlorine oxidative degradation^21^. Our XPS semi-quantitative analysis, shown in the supplementary Table SI and supplementary Figs S7c and S7d, confirms that the MWCNT·PA nanocomposite absorbed substantially lower chlorine ions as compared to pure PA-based membranes.

Another reason for the chlorine resistance of the present nanocomposite membrane is the physical crosslinking effect of MWCNT that might provide not only mechanical reinforcement during water flow operation, but also reduce the degradation avoiding the formation of pinholes. Indeed, PA with a high crosslinking degree is more resistant to degradation in the presence of chlorine^32^. Supplementary Fig. S7b show the XPS study of the membranes before and after the chlorine exposure, and the semi-quantitative analysis is shown in the supplementary Fig. S8. We noticed that unlike pure PA membranes that were prone to fouling due to accumulation of oil traces and iron corrosion products (as identified by XPS - Supplementary Fig. S7a and S8), the MWCNT·PA composite membrane exhibited improved performance against fouling, and almost no iron was detected on the surface after the test. The oil traces (0.4 to 1.6 ppm) and metal ions are common in real processes^33^ because of corrosion, leaks from pumps and gaskets, and seawater contamination, these contaminants are rarely discussed in research studies, but they are particularly common in places where desalination is most needed, such as the Persian Gulf^34^.

The antifouling effect of nanotubes is fundamental since iron impurities have shown to accelerate the chlorine degradation of polyamides^35^. The adhesion of contaminants to the membrane was also studied by AFM on the membrane after cross-flow test, and the results are shown in the supplementary Fig. S9. Chemical analysis of the foulant by ATR and XPS indicate that it is a mixture of oils and ferric products of corrosion which are impurities from our experimental system. After chlorine washing, the foulant is not observed (see Fig. S9b), but the iron content, as indicated by XPS (Supplementary Fig. S7 and Table SI), increased three times, thus confirming that iron diffused into the membrane. Remarkably, the MWCNT·PA-based membrane before and after the chlorine test, exhibited a clean surface (Supplementary Fig. S9c and S9d). In order to evaluate the antifouling properties of our membrane, we exposed the membranes to a 200 ppm of bovine serum albumin (BSA) solution under cross flow with a transmembrane pressure of 1 MPa, and monitored the permeate flow over time. Figure 3d shows the normalized value of the permeate flows of our MWCNT·PA-based membrane, compared to a commercial PA RO membrane and a previously reported MWCNT·PA nanocomposite membrane^7^. The commercial RO membrane shows a typical reduction consistent with constant fouling. In contrast, the fluctuating values displayed in our membrane suggest occasional peeling of the built-up fouling layer followed again by deposition. After 72 h, our membrane still retains 70% of the original flow while the commercial one is only 36%. The spontaneous peeling of the fouling layer observed for the MWCNT·PA membranes might be due to the presence of a smoother surface within the nanocomposite membrane (see Fig. 1c), as well as the inability of the graphitic MWCNT wall to hydrogen bond with the BSA molecules. Interestingly, no changes in desalination performance were observed after the fouling test, and salt rejection rates were higher than 95%. These results suggests that the MWCNT·PA-based RO membrane evidently possess excellent anti-fouling properties in contrast to previous values reported for other MWCNT·PA-based RO membranes with low loading of carbon nanotubes^7^. In that case, a large decline of water flux, as much as 55% of the initial value, was shown after 48 h whereas our membrane showed still higher values after 96 h using a BSA concentration 66 times higher.

Both Raman (Supporting Fig. S10a) and XPS spectra (Fig. S7a) support the enrichment of sp^2^ hybridized carbon after the chlorine exposure. The Raman spectra of the MWCNT·PA nanocomposite shows the characteristic D-band (1345 cm^−1^) and G-band (1600 cm^−1^) of the MWCNT filler (Supporting Fig. S10b). When exposed to chlorine the MWCNT·PA-based nanocomposite exhibited a decrease in the I_D_/I_G_ intensity ratio from 0.81 to 0.52. Similarly, XPS elemental analysis (Table SI) revealed an increase of carbon content from 66.9 at.% to 75.6 at.%, and a ten times decrease in the iron content upon exposure. Therefore, during chlorine water treatment, a sp^2^ hybridized carbon enrichment occurred at the surface, thus forming a protective barrier against chlorine degradation and fouling. Fourier transformed infrared spectroscopy (FTIR) also supports the reduced degradation of the PA·MWCNT nanocomposite membrane (Supporting Fig. S11). After the chlorine water treatment, the pure PA-based membrane exhibited a shift of the amide I band from 1670 cm^−1^ to 1679 cm^−1^ and the peak increased in intensity (Supporting Fig. S11a). The spectral changes indicate that the hydrogen bond interactions were broken between C = O and N-H, thus increasing the non-associated C = O groups. This bond disruption was caused by the electrophilic substitution of N-H by N-Cl and chlorination of the aromatic ring^17^,^36^. When MWCNTs were introduced into the PA membrane, no shift of the amide I group was observed indicating that the MWCNTs prevented the breakage of hydrogen bonds (Fig. S11b). The change was confirmed in the bulk polymers and it is shown in Figs S11c and S11d. The nanocomposite membrane investigated here was compared with other reported nanocomposite RO membranes. Our MWCNT·PA nanocomposite membrane exhibited a far superior permeate flux with salt rejection rates of 90% when 3.5 wt.% NaCl solution and 99.3% when 0.5 wt.% NaCl solution were employed. In order to confirm the chlorine resistance of the MWCNT·PA membrane, both pure PA and the nanocomposite membrane were immersed for 30 min in a 5,000 ppm chlorine solution. The samples were then observed by HRTEM and the images are shown in Fig. S12. The PA membrane changes its color from white to yellow as a result of the chemical degradation. The original PA sample is shown in Fig. S12a, and the sample after chlorination is depicted in Fig. S12b. Although there is not a dramatic change between these images, Fig. S12c shows the appearance of sub 5 nm micropores as a result of membrane degradation. In addition, the MWCNT·PA-based membrane (Fig. S12d) exhibited a different behaviour. We did not find evidence of interfacial degradation between the MWCNT and the PA matrix (Fig. S12e), and pores were not observed in the MWCNT·PA membrane (Fig. S12f). The latter showed greater mechanical stability when exposed to the electron beam during TEM characterization, thus suggesting a more robust and stable structure. Figure 3e and Table SIII compares these values with previously reported desalination values tested with 3.5 wt.% NaCl or lower concentration. Another important aspect for real water desalination applications is the chlorine resistance under active conditions, since it is strongly related to service-life expectancy. Our evaluations on chlorine resistance for membrane containing MWCNT demonstrated an improved resistance to chlorine concentrations up to 4800 ppm·h (see Table SII), which is much better than the reported resistance for other MWCNT·PA-based membranes with lower MWCNT loading. The permeate flux was stable and the membrane exhibited an outstanding structural resistance against pinholes produced by chlorine.

In summary, RO nanocomposite membranes containing non-covalently functionalized MWCNT were successfully prepared by interfacial polymerization. The active layer of the nanocomposite membrane consisted of aromatic polyamide with a large fraction of well dispersed MWCNT that promoted a flux-enhanced microstructure around the nanotubes surface. We found that the addition of MWCNT decreased iron adsorption in the PA, and kept the surface of the membranes free from fouling, and most importantly, provided the RO membrane with an outstanding chlorine resistance. Thus, the nanocomposite MWCNT·PA-based membranes not only exhibited high desalination membrane performance in terms of 1) high rejection rate, high flow and 2) antifouling nature, but also 3) inhibited the attack by chlorine at the expense of a moderate reduction in salt rejection. Taking into account the differences in NaCl concentration, the membranes prepared in this work had a higher salt rejection and higher permeate flux, demonstrating an outstanding performance when compared to other published works. Such high performance can be attributed to the characteristic structure established around the MWCNT within the PA matrix. The present nanocomposite RO membrane using MWCNT, a commercially available nanomaterial, can be considered a breakthrough and can become a promising candidate for next technological platform in water purification in the “Century of Water”.

## Experimental Part

### Materials

m-Phenylenediamine(>98%) (MPD) and trimesoyl chloride (>98%) (TMC) were acquired from *Tokyo Chemical Industry Co. Inc.* Hexane (>96%), sodium Chloride (>99.5%), and sodium hypochlorite solution (5.0 wt.%) were purchased from *Kanto Chemical Co., Inc.* (Purity>96%). All other reagents were of analytical grade or better. Surfactant dispersed MWCNT (*Nanocyl NC-7000*) were prepared as reported^37^. Polypropylene nonwoven supported porous polysulfone membranes (cut off Mw = 100,000) were acquired from *Alfalaval DSS* (Cat. No. GR40PP). We confirmed that the water permeability of the present thin-film nanocomposite membranes did not change due to compaction at pressures up to 5 MPa using 3.5 wt. % saline water at room temperature, as described in the following experimental methods.

### Methods

#### Membrane synthesis

composite membranes consisting of porous polysulfone supported on nonwoven polypropylene (*Alfa Laval* ) were soaked in 2.0 wt. % MPD aqueous solution for 3 h and subsequently soaked in TMC-hexane solution (0.1 wt. %) for 2 min. The membranes were subsequently dried at room temperature for several hours. In the case of the MWCNT containing PA membrane, a MPD aqueous solution containing 1 wt.% of anionic surfactant stabilized MWCNT was used. In order to study the polymerization in presence of carbon nanotubes, an aqueous dispersion of MWCNT was added to a MPD aqueous solution to reach a 0.4/2.0/97.6 MWCNT/MPD/H_2_O ratio and weight. Then, the solution was filtered, and the cake was re-dispersed in hexane by stirring and ultrasonication, stirred for 1 h and filtered again. These MWCNT were re-dispersed in hexane and then a small volume of this dispersion was added to a 0.1% solution of TMC in hexane. After 1 h the nanotubes were recovered by centrifugation, washed in hexane and analysed by HRTEM, XPS, FTIR and TGA. Bulk PA and MWCNT·PA samples were synthesized by interfacial polymerization of a 2% wt MPD solution in water and a 0.1% wt solution of TMC in hexane. Degradation by chlorine was also studied by exposing the bulk PA and MWCNT·PA samples to (200 ppm for 24 h ) and (5000 ppm for 30 min) chlorine solution at room temperature, followed by washing and drying. Fouling tests were carried out using room temperature crossflow filtration of an aqueous Bovine serum albumin (BSA) solution (200 ppm) at 1 MPa.

#### Membrane characterization

Thermogravimetric/differential thermal analysis was conducted on a *TGA 8120* apparatus under flowing air (300 mL/min) and at a heating rate of 10 ^°^C/min; alumina powder was used as a reference material. **AFM** studies were performed in an Agilent 5500 atomic force microscope using Al coated silicon tips at 139 kHz resonance frequency. Topography and phase images were simultaneously acquired and the images were contrast enhanced using the *Gwyddion* software. X-ray photoelectron spectroscopy **(XPS)** analysis was performed using the Al-Kα line in an *Axis-Ultra, Kratos*, UK. The XPS analysis chamber was operated at 10^−9^ Torr, the X-ray beam was focused in a 700 μm × 300 μm area, and the analyser was set at 160 and 20 eV pass energy for the survey and narrow scan data acquisition, respectively. Semi-quantitative chemical analysis was performed by integrating the C 1 s, O 1 s, N 1 s, Fe 2p, and Cl 2p peaks from survey scans. Samples were adhered to double-sided carbon tape, grounded and charge neutralized with a low-energy electron gun. Samples were referenced to the C 1 s sp^3^ peak at 285.1 eV. **Raman spectroscopy** was conducted in a *Renishaw* micro-Raman using the 532 nm excitation line. Mapping was performed by acquiring a 256 × 256 spectra over a 30 × 30 μm^2^ grid. Fourier-transform infrared (**FTIR**) spectroscopic studies were conducted on a *Nicolet 6700* spectrophotometer operated in attenuated total reflectance mode. Membrane surfaces and fractured cross-sections were examined by optical (Olympus BX-51 equipped with a digital camera) and scanning electron microscopy SEM (JEOL JSM-6335 operated at 15 kV). High resolution transmission electron microscopy was conducted in a Cs corrected JEOL JEM-2100 F microscope operated at 80 kV of acceleration voltage.

#### Monomers structural simulation

monomers preferred orientation in the vicinity of a sp^2^ hybridized hexagonal network was performed using the Avogadro molecular modeling software. To specify the characteristic structure developed in the present materials, the energies for each orientation were calculated after geometrical optimization of each system. The calculations were performed under the framework of the Universal Force Field (UFF) method, with optimization using a steepest descent algorithm and convergence energy criteria of 10^−9^ KJ mol^−1^. The cohesion energy calculated for each systems corresponds to the graphene molecule non-covalent interaction stability, calculated as:
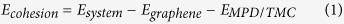
where *E*_*cohesion*_ is the energetic stability, *E*_*system*_ is the energy of each system, *E*_*graphene*_ is the graphene section energy, and *E*_*MPD/TMC*_ is the energy of the corresponding molecule.

For molecular dynamics of MPD and TMC adsorption on graphene and single walled carbon nanotube surfaces, MD simulations were performed with DL_POLY package, under the NVT ensemble at 300 °K using Nosé-Hoover algorithm for temperature control. The DREIDING force field was used for modelling MPD and TMC molecules dynamics^37^. The interactions between MPD and TMC (MPD and MPD, TMC and TMC) are calculated by Lenard-Jones (LJ) and Coulomb potentials. The atom charges of MPD and TMC are determined by ab initio quantum chemistry calculation at MP2/6-31 G** level, using NWCHEM code. The interactions between SWCNT and graphene surfaces with MPD or TMC are calculated by LJ potential only. All LJ parameters were referenced to^38^. SWCNT or graphene interaction are described by Tersoff potential^39^. The simulations used a timestep of 0.1 fs, with a complete simulation time of 1 ns for statistical analysis. All cutoff radii were set to 15.0 Å The simulated components were an armchair SWCNT (10,10) with diameter of 13.56 Å, a graphene sheet with 840 atoms, and 50 MPD molecules located randomly for initial stage. After MPD adsorption, TMC molecules were located randomly around SWCNT and graphene (40 and 20 molecules, respectively), for co-adsorption and structural arrangement study.

#### Membrane desalination tests

The desalination performance of PA RO and MWCNT·PA nanocomposite membranes were evaluated in a modified automatized system for cross flow experiments (*GE Osmonics*). The membranes were loaded in a stainless steel circular cross-flow cell (circular shape, 2.5 cm diameter) and pressurized up to 5 MPa while testing feed water solutions with 3.5, 0.5 and 0.2 wt.% NaCl. The permeate flow was evaluated gravimetrically and its salinity was evaluated by conductimetry.

## Additional Information

**How to cite this article**: Inukai, S. *et al.* High-performance multi-functional reverse osmosis membranes obtained by carbon nanotube·polyamide nanocomposite. *Sci. Rep.* **5**, 13562; doi: 10.1038/srep13562 (2015).

## Supplementary Material

Supplementary Information
