# Supplementary material for: High-performance multi-functional reverse osmosis membranes obtained by carbon nanotube·polyamide nanocomposite
Source: Sci Rep. 2015 Sep 3;5:13562. doi: 10.1038/srep13562 (PMC4558580; doi:10.1038/srep13562)
Supplement: Supplementary Information [file srep13562-s1.pdf]

Supplementary information

High-performance multi-functional reverse osmosis membranes obtained by carbon nanotube·polyamide nanocomposite.

Shigeki Inukai,[1] Rodolfo Cruz-Silva,[1] Josue Ortiz-Medina,[1] Aaron Morelos-Gomez,[1] Kenji Takeuchi,[1,2] Takuya Hayashi,[1,2] Akihiko Tanioka,[2] Takumi Araki[1,3], Syogo Tejima[1,3], Toru Noguchi,[1,2] Mauricio Terrones,[2,4] and Morinobu Endo.[1,2,*]

[1]Global Aqua Innovation Center, Shinshu University; 4-17-1 Wakasato, Nagano 380-8553, Japan.

[2]Institute of Carbon Science and Technology, Shinshu University; 4-17-1 Wakasato, Nagano 380-8553, Japan.

[3]Division of Computational Science and Technology, Research Organization for Information Science & Technology, Shinagawa, Tokyo, Japan

[4]Department of Physics, Department of Materials Science and Engineering and Department of Chemistry. The Pennsylvania State University; University Park, Pennsylvania 16802, USA.

*Author correspondence: endo@endomoribu.shinshu-u.ac.jp

Table SI. XPS semi-quantitative chemical analysis of PA and MWCNT·PA RO membranes and their bulk polymers before and after chlorine water exposure (200 ppm for 24h). Membranes were also tested for desalination with 3.5 wt % NaCl solution for 2h.

**Membranes**	Element (atomic %)				
	C	O	N	Cl	Fe
PA before Cl exposure	74.0	16.9	8.4	0.6	0.2
PA after Cl exposure	68.8	20.2	7.2	3.2	0.6
MWCNT·PA before Cl exposure	66.9	20.5	8.1	3.5	1.0
MWCNT·PA after Cl exposure	75.1	14.9	9.4	0.5	0.1
**Bulk polymers**	C	O	N	Cl	Fe
PA before Cl exposure	77.1	8.9	14.1	0.0	0.0
PA after Cl exposure	74.9	9.3	12.1	3.8	0.0
MWCNT·PA before Cl exposure	79.4	9.7	10.9	0.0	0.0
MWCNT·PA after Cl exposure	77.7	10.6	9.4	2.3	0.0

Table SII. Chlorine resistance under active chlorine capacity conditions shown by the nanocomposite MWCNT·PA-based membranes compared with previous work by Zhao et al. (*7*) Data have been normalized considering the initial flow and desalination rate as 100 %. The present membrane has a more stable performance than others.

**Chlorine (ppm·h)**	**Normalized salt rejection (%)**			**Normalized water flux (%)**		
	PA	MWCNT·PA	MWCNT·PA (Our work)	PA	MWCNT·PA	MWCNT·PA (Our work)
0	100	100	100	100	100	100
2400	n.a.	n.a.	100	n.a.	n.a.	100
2500	88	98	n.a.	220	110	n.a.
3500	84	97	n.a.	250	120	n.a.
4800	n.a.	n.a.	100	n.a.	n.a.	110

n.a. = not available

Table SIII Comparison of combined rejection rate/permeate flux performance of the nanocomposite MWCNT·PA membranes studied in this work and previous literature values of other nanocomposite membranes (only membranes with rejection rates higher than 90 % in the reported papers were considered).

Reference (Filler, wt %)	Rejection %	Flow LMH	Pressure bar	$m^3/m^2\,d{\cdot}MPa$	NaCl wt%
Duan et al. [1] POSS-PA (0.4%)	98.9	27.1	15.5	0.42	0.2
Kim et al.[2] CNT-PA (0.17%)	95.7	44.3	15.5	0.69	0.2
Zhao et al. [3]MWCNT-PA (0.1%)	90.0	28.1	16.0	0.42	0.2
Chan et al. [4] SWCNT-PA (20 %)	98.6	48.4	36.5	0.32	0.2
LFC-1 (polyamide TFC) [2]	96.5	39.5	15.5	0.61	0.2
Lee et al.(PDOPA- MWCNT-PA)[5]	98.5	52.8	15.5	0.82	0.2
GO-Polyamide[6]	96.4	14.0	15.5	0.22	0.2
Inukai et al. (This work. MWCNT·PA)	99.8	90.4	15.5	1.40	0.2
Inukai et al. (This work. MWCNT·PA)	90.0	79.2	50.0	0.38	3.5
Inukai et al. (This work. MWCNT·PA)	99.3	312.5	50.0	1.50	0.5

Table references

1 Duan, J. T., Litwiller, E. & Pinnau, I. Preparation and water desalination properties of POSS-polyamide nanocomposite reverse osmosis membranes. *Journal of Membrane Science* **473**, 157-164, doi:10.1016/j.memsci.2014.09.022 (2015).

2 Kim, H. J. *et al.* High-Performance Reverse Osmosis CNT/Polyamide Nanocomposite Membrane by Controlled Interfacial Interactions. *Acs Applied Materials & Interfaces* **6**, 2819-2829, doi:10.1021/am405398f (2014).

3 Zhao, H. Y. *et al.* Improving the performance of polyamide reverse osmosis membrane by incorporation of modified multi-walled carbon nanotubes. *Journal of Membrane Science* **450**, 249-256, doi:10.1016/j.memsci.2013.09.014 (2014).

4 Chan, W. F. *et al.* Zwitterion Functionalized Carbon Nanotube/Polyamide Nanocomposite Membranes for Water Desalination. *Acs Nano* **7**, 5308-5319, doi:10.1021/nn4011494 (2013).

5 Lee, H. D., Kim, H. W., Cho, Y. H. & Park, H. B. Experimental evidence of rapid water transport through carbon nanotubes embedded in polymeric desalination membranes. *Small (Weinheim an der Bergstrasse, Germany)* **10**, 2653-2660, doi:10.1002/smll.201303945 (2014).

6 Choi, W., Choi, J., Bang, J. & Lee, J. H. Layer-by-Layer Assembly of Graphene Oxide Nanosheets on Polyamide Membranes for Durable Reverse-Osmosis Applications. *Acs Applied Materials & Interfaces* **5**, 12510-12519, doi:10.1021/am403790s (2013).

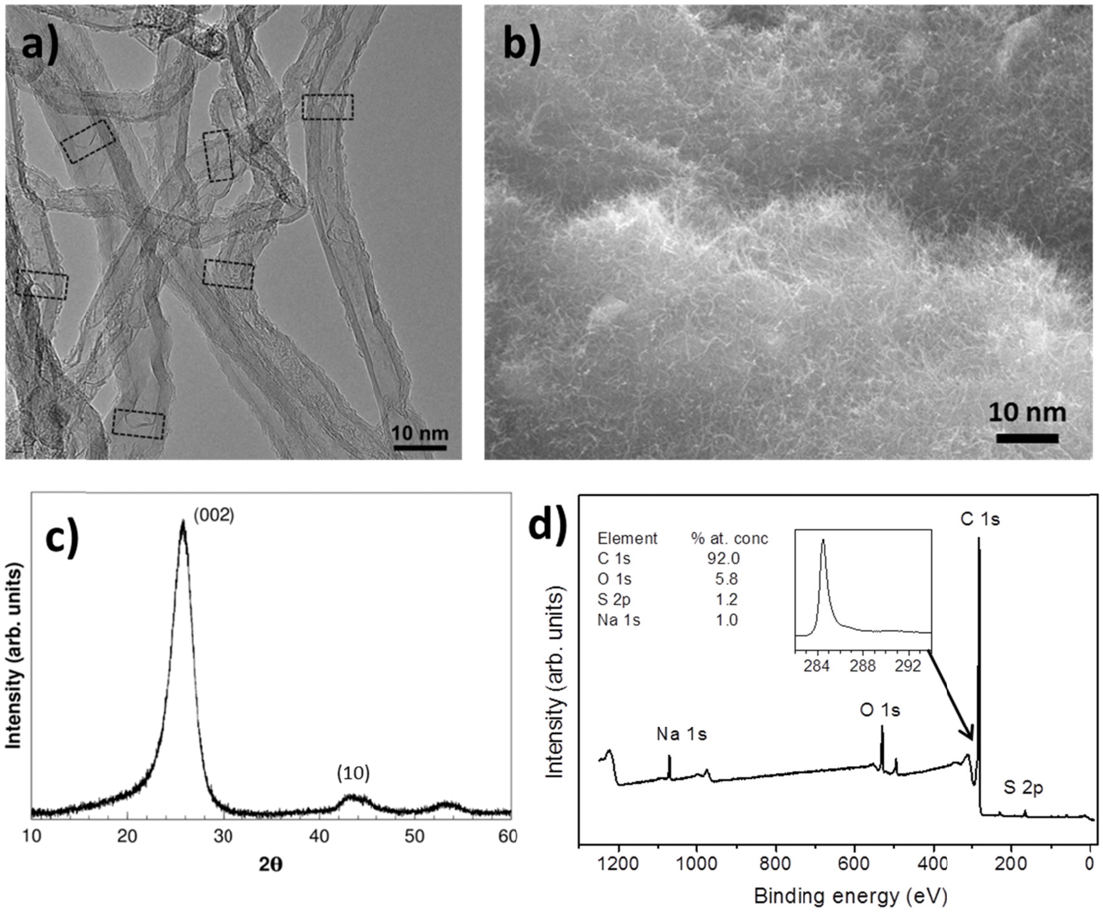

Figure S1. a) HRTEM image of pristine MWCNT used in this work. "Bridge" structures inside the nanotube, preventing water flow in the hollow core, are clearly visible in the present samples, and indicated with dotted boxes in the figure; b) SEM image of the Nanocyl-700 MWCNT; c) the X-ray diffraction pattern of Nanocyl-7000 dispersion showing the characteristic (002) spacing and the (10) peaks of turbostratic carbon, and d) X-ray photoelectron spectroscopy and semi-quantitative elemental analysis of the carbon nanotubes. With 92 % of atomic carbon and 1.2 % of sulphur, the MWCNTs exhibit relatively clean walls for a water dispersible material.

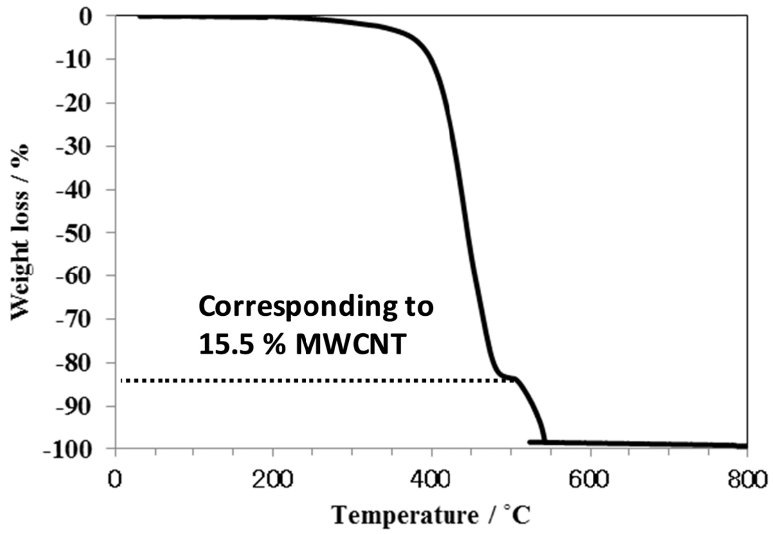

Figure S2. Thermogravimetric analysis of the MWCNT·PA nanocomposite reverse osmosis membrane. Approximately 15.5 wt.% of MWCNT was entrapped in the MWCNT·PA-based membrane.

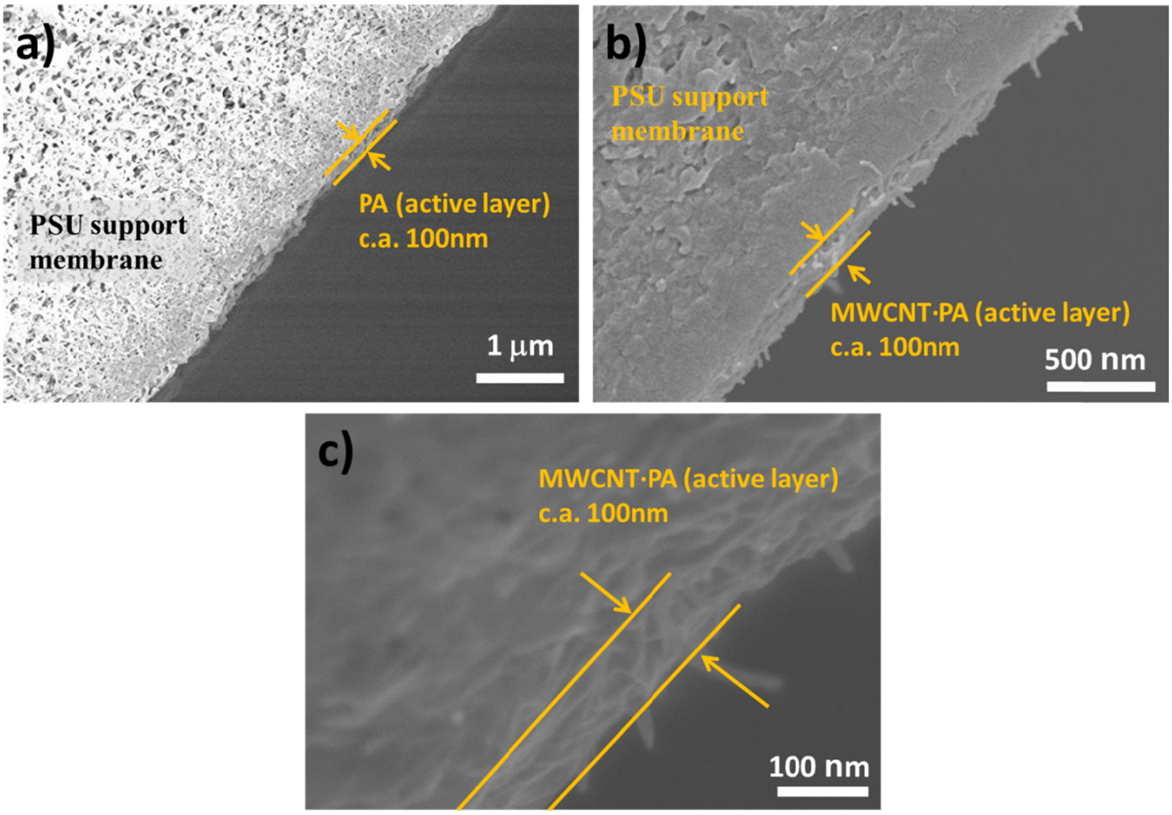

Figure S3. SEM images of the cross sections of the freeze-fractured PA-based membrane and the composite RO membranes synthesized. a) PA-based membrane on porous polysulfone; b) MWCNT·PA-based membrane on top of porous polysulfone, and c) high magnification image of the nanocomposite active layer cross section indicating CNT fillers.

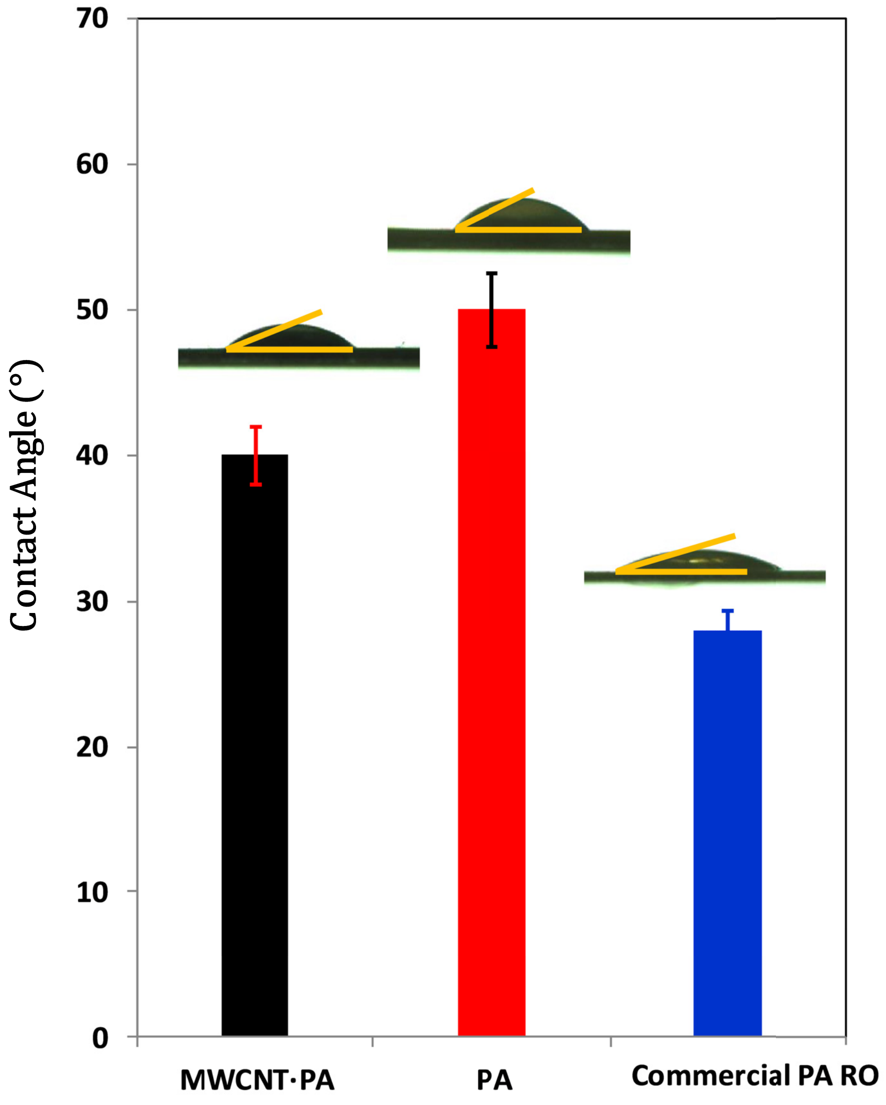

Figure S4. Static water contact angle of different membrane samples studied.

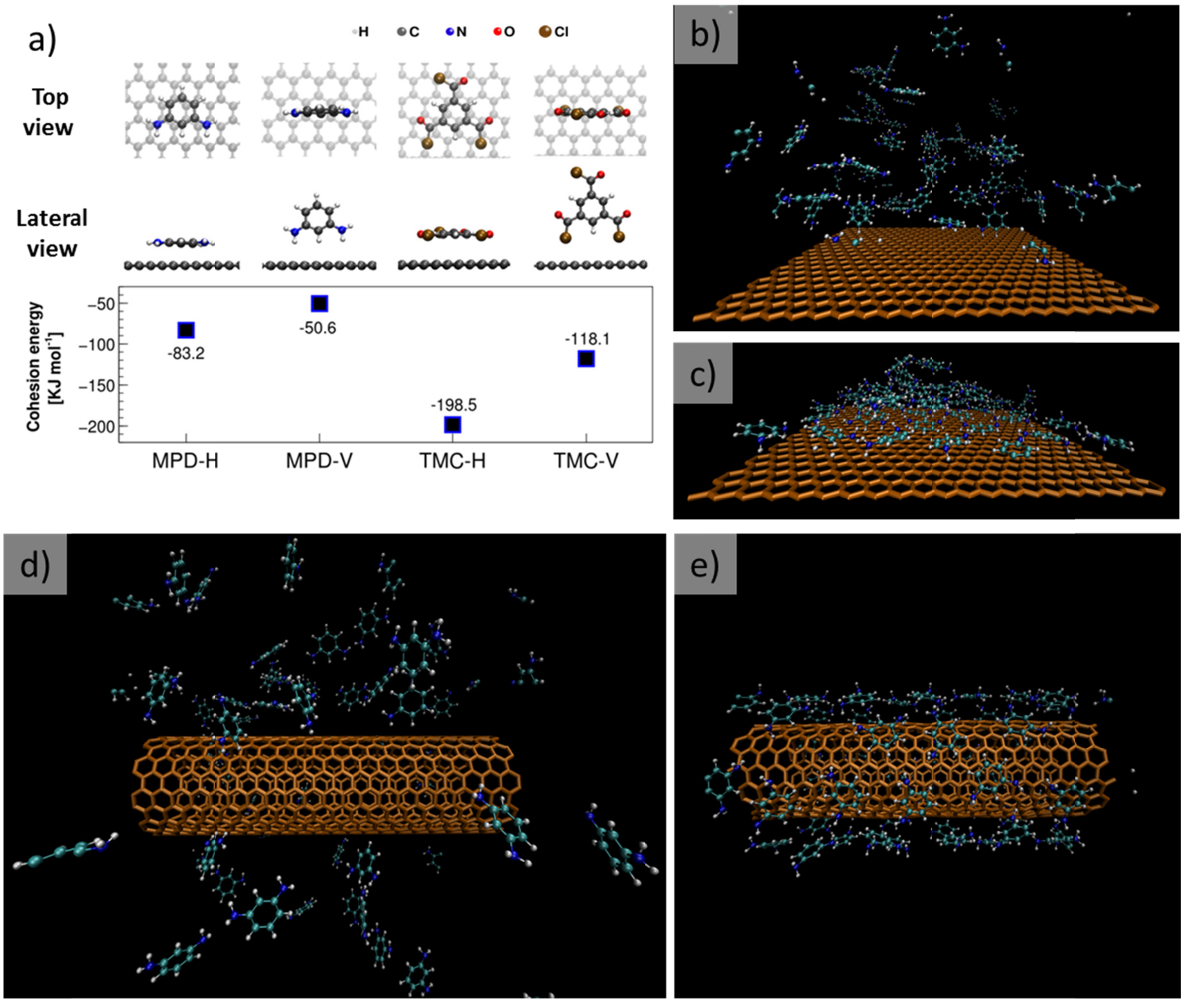

Figure S5. Energetic stability of relaxed simulated models of MPD and TMC molecules adsorbed on graphene surface, which can be considered equivalent to MWCNT surface. The top molecular models depict the top and side view of simulated systems: a) MPD and TMC parallel (MPD-H) and perpendicular (MPD-V) with respect to network surface; b) Shows the initial and c) relaxed state of several MPD molecules on a graphene surface. Most MPD molecules rearranged parallel to the surface. Similar results were obtained when modelling adsorption of MPD molecules on a single walled carbon nanotube: d) initial state and e) relaxed state.

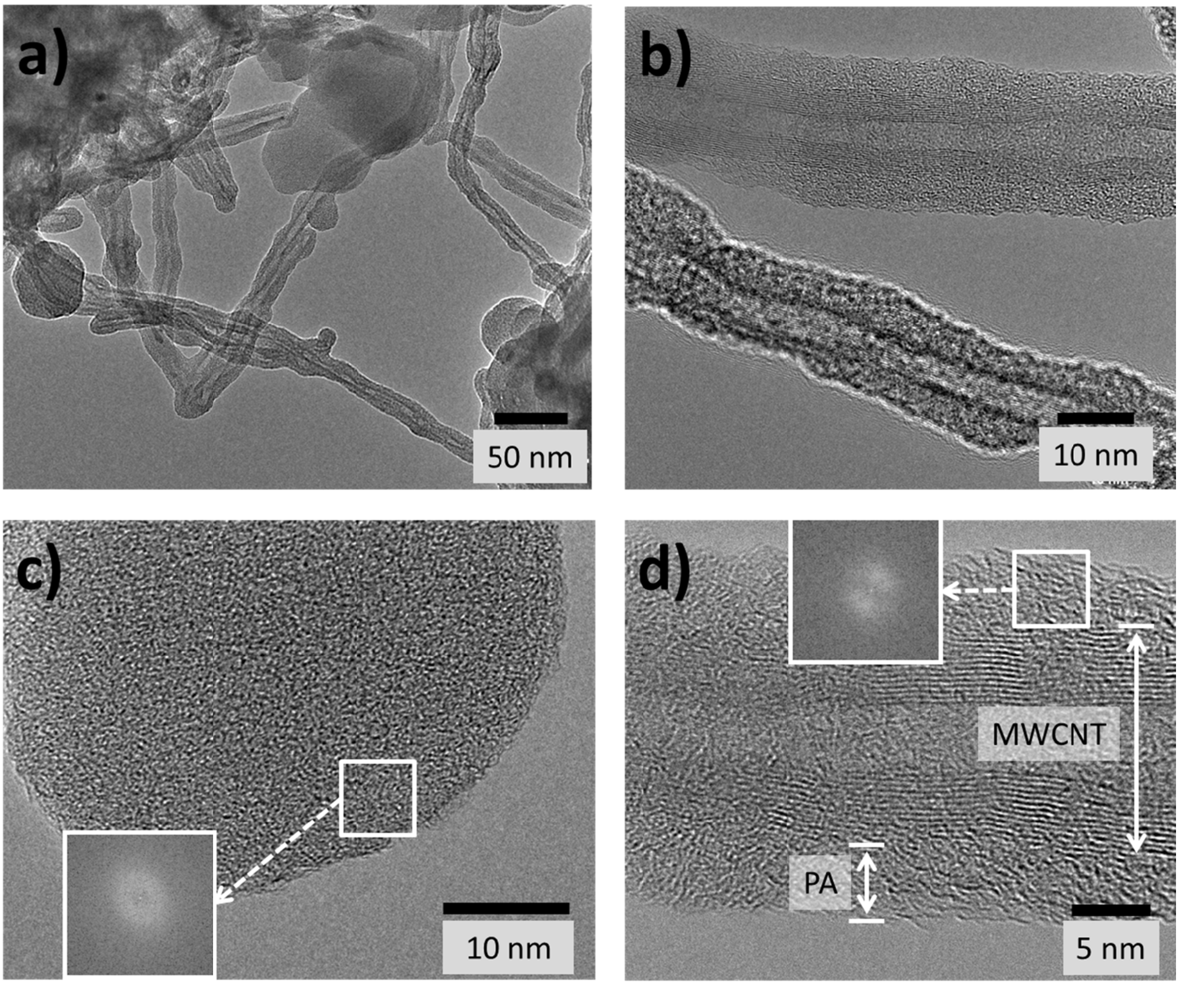

Figure S6. High resolution transmission electron microscopy study of the PA coated MWCNT. a) MWCNT showing coating by PA. Diameter of the coated MWCNT is approximately 20 nm, two times the diameter of the pristine nanotubes; b) shows a higher magnification of the nanotubes showing a homogeneous coating by PA; c) FFT analysis of the pure PA particles shows an homogenous halo FFT pattern characteristic of disordered amorphous networks, whereas in d) the FFT pattern of the PA coating suggest preferential orientation of the atomic network in a direction parallel to the surface of the MWCNT.

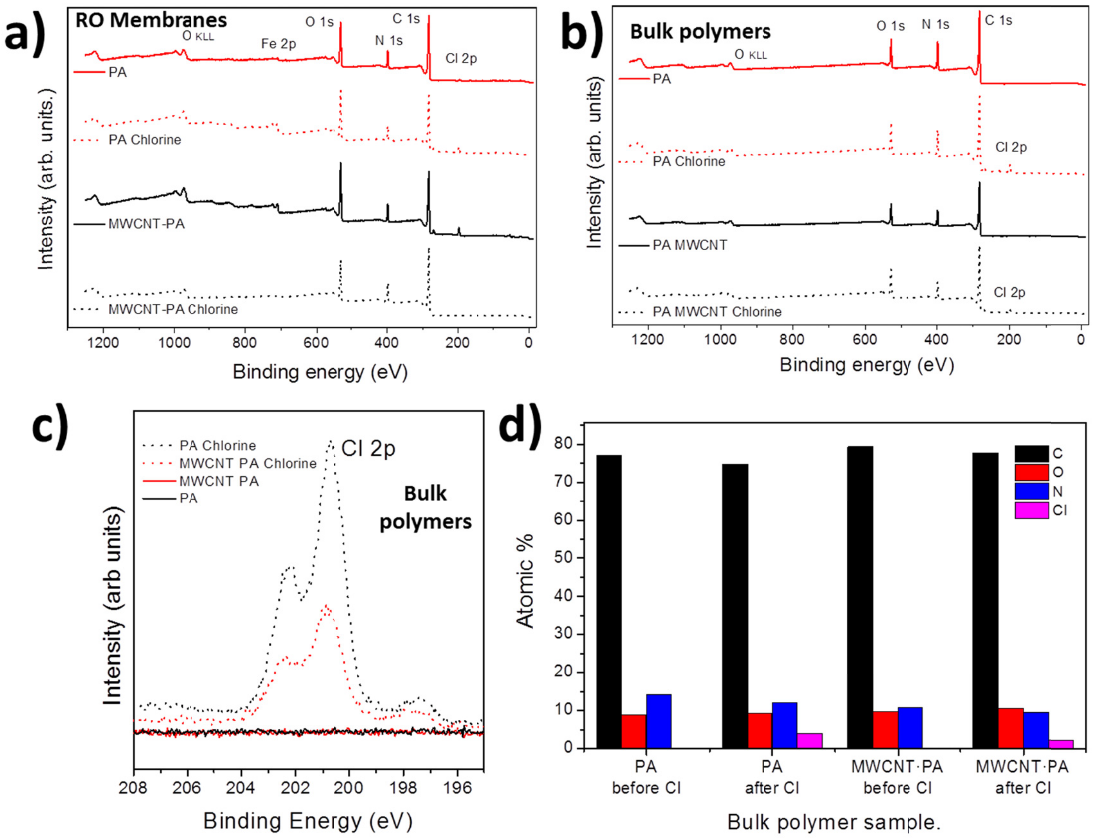

Figure S7. XPS elemental analysis of a) pure PA and MWCNT·PA reverse osmosis membranes before and after exposure to chlorine water (200 ppm 24 h); b) shows similar wide scan spectra obtained from bulk synthesized PA and MWCNT·PA, and c) shows the narrow scan of the 2p peak chlorine and d) elemental analysis of the bulk polymers before and after chlorine water exposure test (200 ppm, 24 h).

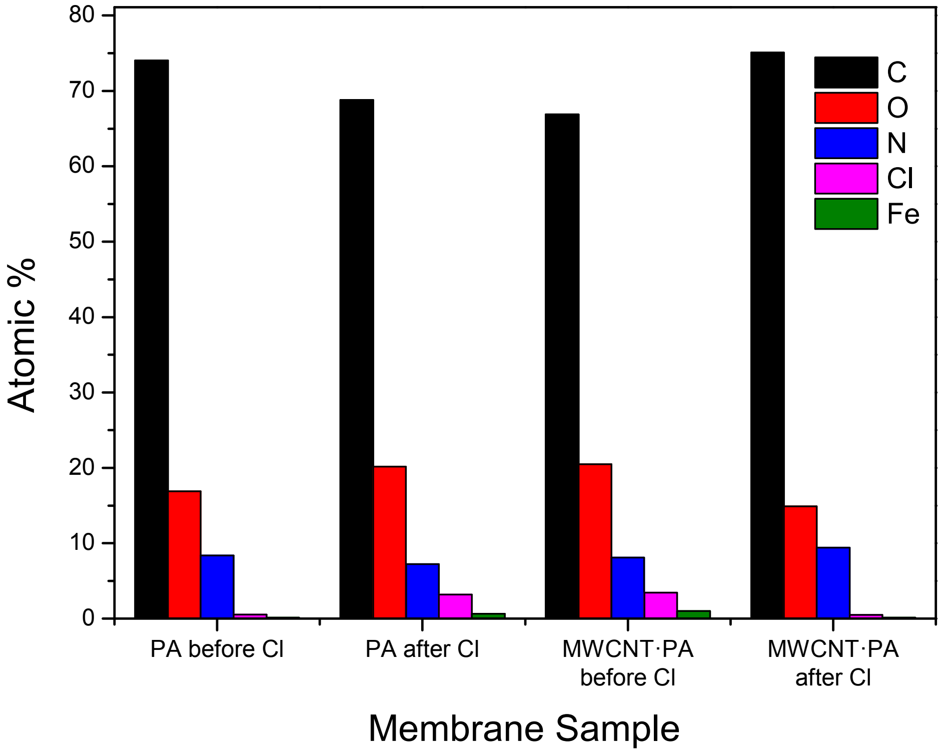

Figure S8. Elemental analysis from XPS of the pure PA and MWCNT·PA nanocomposite reverse osmosis membranes showing before and after chlorine water exposure (200 ppm, 24 h). Membranes were previously tested in desalination test in 3.5 wt % NaCl for 2 h.

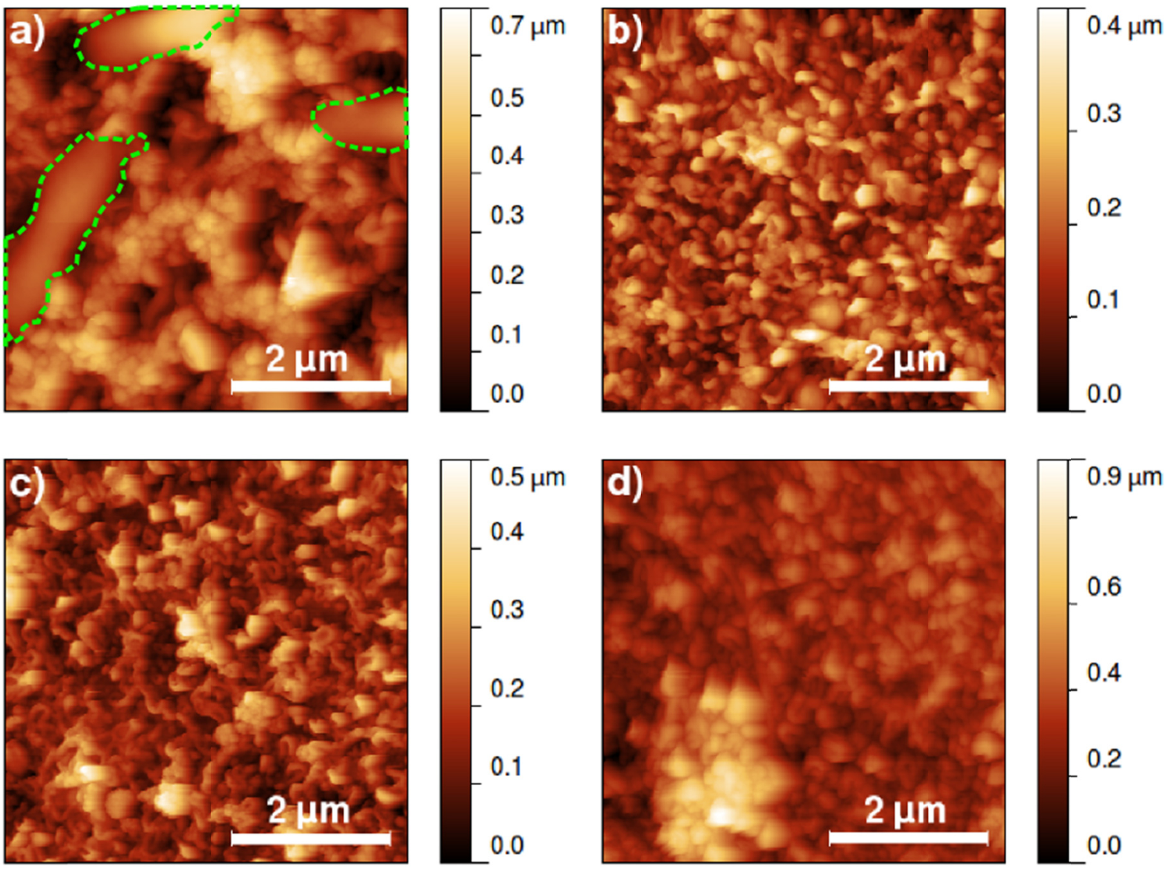

Figure S9. AFM topography images of the PA active layer of the nanocomposite membranes. a) original pure PA, indicating contaminant foulant deposited on the surface; b) PA after chlorine water exposure (200 ppm, 24 h) showing cleaned surface; c) MWCNT·PA-based nanocomposite layer with no deposited fouling layer, and d) MWCNT·PA-based nanocomposite layer after chlorine water exposure showing no change as original surface. All samples were imaged after testing in NaCl 3.5 % wt solution at 5 MPa for 2h.

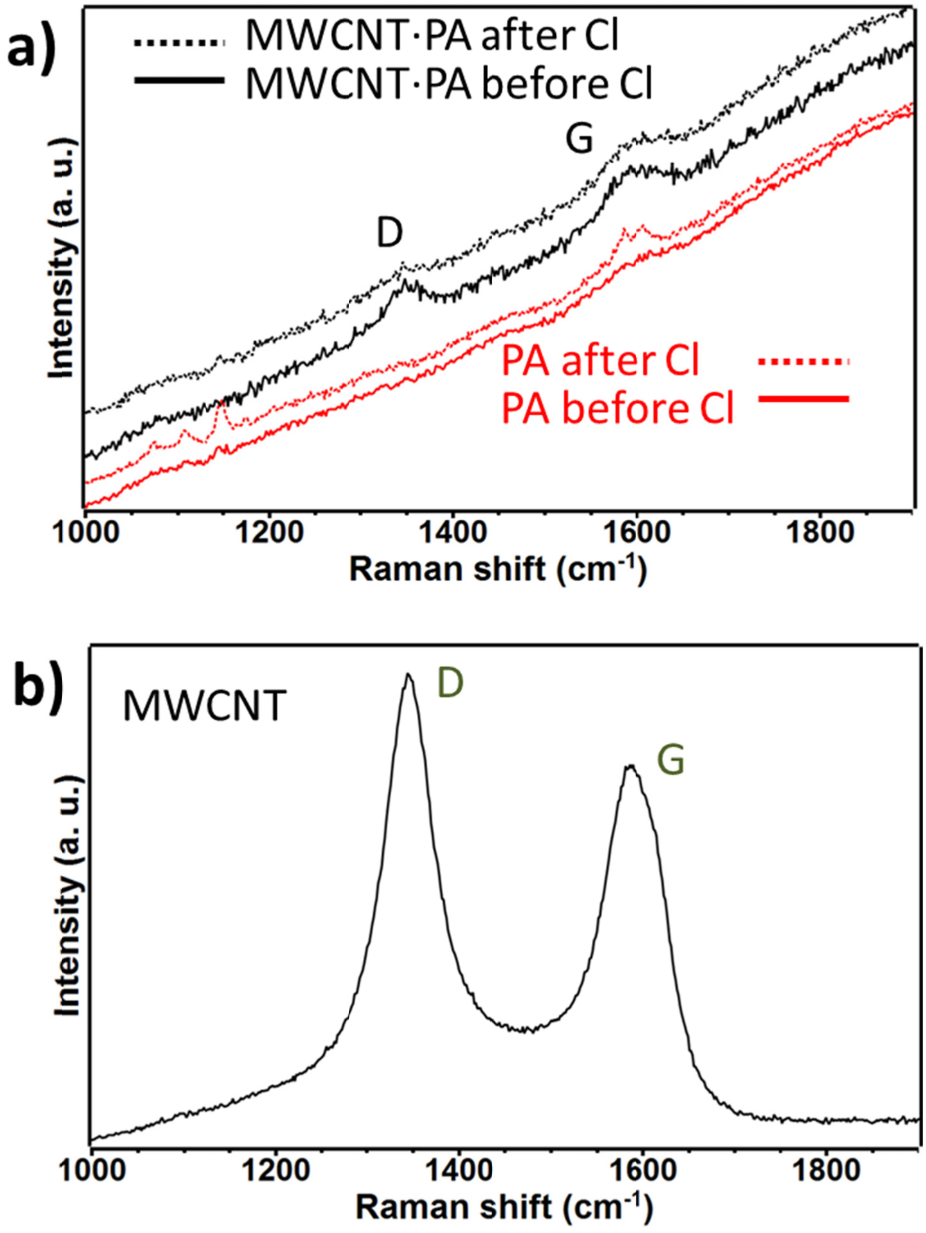

Figure S10. a) Raman spectra of pure PA and MWCNT·PA nanocomposite RO membranes before and after exposure to chlorine water (200 ppm, 24 h), and b) pristine MWCNT. There are greater differences for MWCNT·PA membrane whereas the pure PA membranes before and after chlorine exposure exhibit small changes. Exposure to chlorine water leads to changes between 1070 cm^{-1} and 1150 cm^{-1} and at 1600 cm^{-1}. The peaks that are between 1070 cm^{-1} and 1150 cm^{-1} reflect the C-N stretching vibration modes from amide groups. In addition, the peaks around 1600 cm^{-1} correspond to aromatic amide groups.

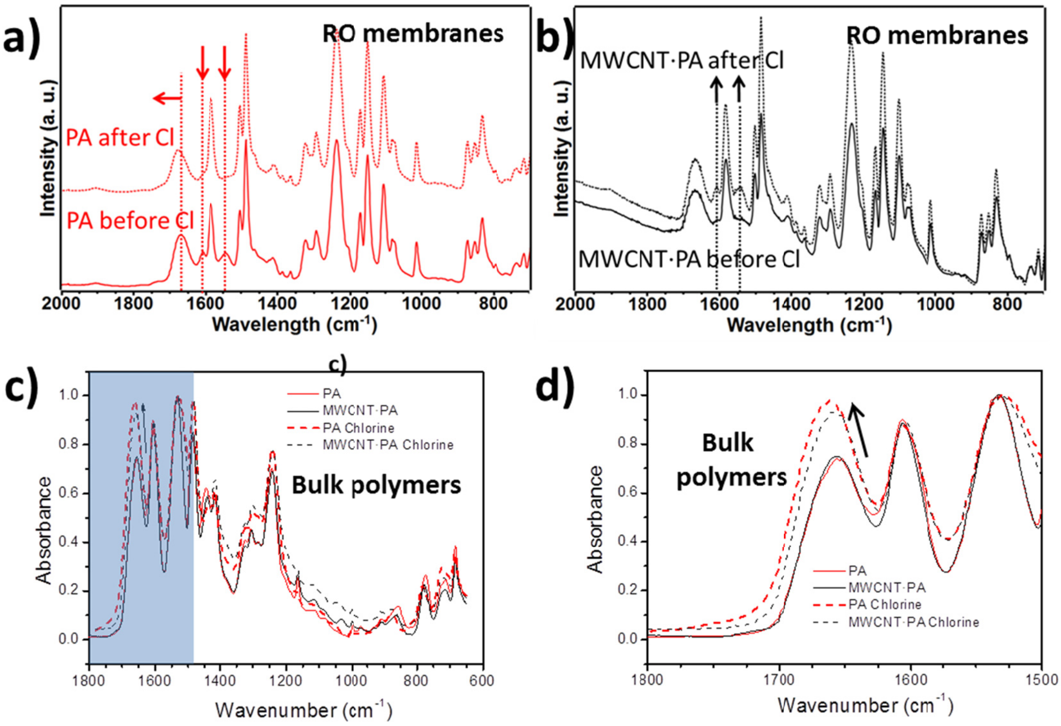

Figure S11. Attenuated total reflectance-FTIR spectra of the a) PA membranes and b) MWCNT·PA nanocomposite membranes before and after exposure to chlorine water. While the peaks are dominated by the porous polysulfone support, small changes in amide peaks are visible; c) Spectra of the as-prepared pure PA and MWCNT·PA bulk polymers are shown, and d) the shaded area in the proximities of the amide I band in c) is shown by enlarging.

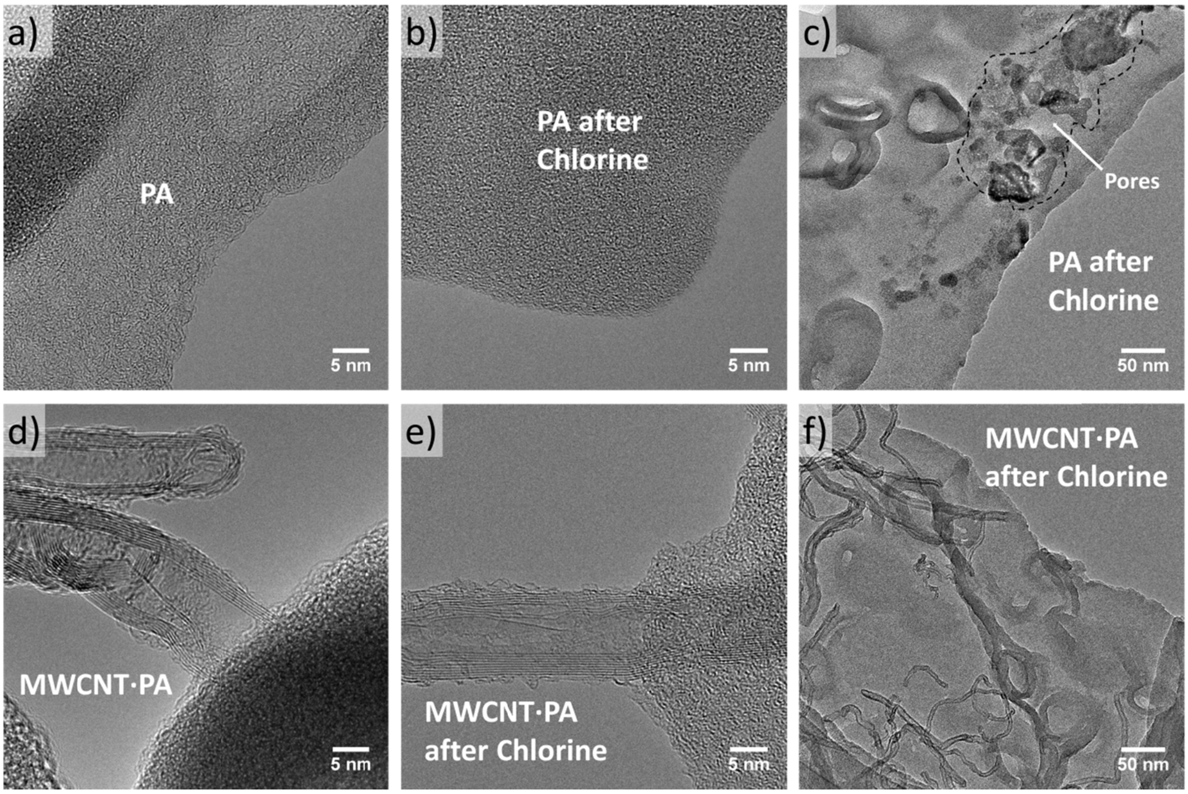

Figure S12. HRTEM study of bulk PA and MWCNT·PA samples after strong chlorination treatment (exposure to chlorine water at 5000 ppm for 30 min). a) Image of the original pure PA sample; b) Image of PA after chlorination; c) Low magnification image of PA membrane, notice the formation of small pores (sub 5 nm diameter); d) Image of the original MWCNT·PA membrane; e) Image of the MWCNT·PA membrane after chlorination, showing good interfacial adhesion between the PA matrix and the MWCNT; f) Low magnification image of MWCNT·PA membrane. No pore formation was observed and the membrane showed higher stability under the electron beam irradiation as compared with the chlorine treated pure PA membrane shown in c.
